# Association of Polymorphisms of the Receptor for Advanced Glycation End Products Gene and Susceptibility to Sporadic Abdominal Aortic Aneurysm

**DOI:** 10.1155/2015/394126

**Published:** 2015-02-18

**Authors:** Ye Yao, Junli Zhuang, You Li, Bao Jing, Hali Li, Jingbo Li, Changgang Shao, Keshen Li, Haiyang Wang

**Affiliations:** ^1^Division of Vascular Surgery, Department of General Surgery, The First Affiliated Hospital of Harbin Medical University, Harbin, Heilongjiang 150001, China; ^2^Guangdong Key Laboratory of Age-Related Cardiac and Cerebral Diseases, Affiliated Hospital of Guangdong Medical College, Zhanjiang 524001, China

## Abstract

Accumulating evidence has suggested that receptor for advanced glycation end products (RAGE) is involved in the development and progression of human abdominal aortic aneurysms (AAAs). However, the association between* RAGE* gene polymorphisms and AAA has not yet been determined. The present study was aimed at analyzing the potential association between the* RAGE* gene polymorphisms and AAAs. A cohort of 381 patients and 436 age-matched healthy controls were genotyped to detect the three* RAGE* polymorphisms (−374 T/A, −429 T/C, and G82S) using SNaPshot. Our study demonstrated a significant difference in the genotype and allele frequencies of the* RAGE* G82S polymorphism between the AAA patients and the controls. Further stratification by gender and smoking status revealed that the presence of the* RAGE* 82S allele confers a higher risk for developing AAA in men and smokers. Moreover, AAA patients with the variant 82S allele of* RAGE* presented with reduced serum soluble RAGE (sRAGE) production, and this decrease was more significant in men and smokers with AAA. Our study provides preliminary evidence that the 82S allele of* RAGE* is a risk factor for AAA. This new piece of knowledge regarding RAGE may be clinically important for the prevention and therapy of AAAs.

## 1. Introduction

Human abdominal aortic aneurysm (AAA), a major disease affecting the aorta, is the most common type of aneurysm in humans [1]. Rupture of the AAA is associated with high mortality, making aortic aneurysms a leading cause of death. The etiology of AAA involves chronic inflammation [2], loss of the extracellular protein elastin [3], increased neoangiogenesis [4], enhanced oxidative stress [5], and excessive degradation of the extracellular matrix (ECM) [6]; nevertheless, the precise mechanisms of AAA formation and progression are still elusive [7].

Receptor for advanced glycation end products (RAGE), a member of the immunoglobulin (Ig) superfamily, can interact with a broad range of ligands, including advanced glycation end products (AGEs), S100/calgranulins, high mobility group box-1 (HMGB1) protein, and *β*-amyloid peptide [8]. The binding of RAGE to its ligands not only stimulates the generation of reactive oxygen species (ROS) and the activation of nuclear factor kappa-B (NF-*κ*B), mitogen-activated protein kinase (MAPK), and protein kinase C (PKC) but also induces cytokine production and inflammatory reactions, all of which are involved in the development and progression of AAAs [9]. In contrast, the soluble form of RAGE (sRAGE) can potentially bind to an AGE ligand thereby acting as a decoy, preventing the adverse effects of RAGE signaling [10]. The expression of RAGE and its ligand AGE was found to be significantly upregulated in human aneurysm tissues [11].* In vivo *evidence also demonstrated that the inhibition of AGE signaling via targeted gene deletion of RAGE dramatically reduced the incidence of AAA in a mouse model [11]. Moreover, the aortic diameter was markedly abated in RAGE-deficient animals [11]. These lines of evidence have led us to hypothesize that RAGE may play a significant role in the pathogenesis of AAA.

The gene encoding* RAGE *is located in the major histocompatibility complex locus on chromosome 6p21.3, and a number of polymorphisms have been identified [12]. The three most extensively studied polymorphisms include two SNPs in the promoter region (−429 T/C and −374 T/A) and one SNP in exon 3 (G82S) of the* RAGE* gene. The −429 T/C and −374 T/A polymorphisms have been shown to exert significant effects on the transcriptional activity [13], and the G82S polymorphism, which occurs in the AGE binding domain, has been shown to have an enhanced ligand-binding affinity and leads to the increased ligand-stimulated activation of proinflammatory mediators [14]. The functional polymorphisms of the* RAGE* gene have been studied for their associations with various diseases, including Crohn's disease, systemic lupus erythematosus, Alzheimer's disease, multiple sclerosis, and cardiovascular diseases [15–20]. However, the association between the* RAGE *polymorphisms and AAA has not yet been determined. In light of the significant role of RAGE in the pathogenesis of AAA, the present study aimed to investigate the association of the* RAGE* polymorphisms (−374 T/A, −429 T/C, and G82S) with AAA in a case-control study of the Han Chinese population.

## 2. Materials and Methods

### 2.1. Study Population

Our study consecutively recruited 381 patients (292 males and 99 females) with AAA from the Department of Vascular Surgery at the First Affiliated Hospital of Harbin Medical University from 2004 to 2013. The AAA diagnosis was confirmed using ultrasound scanning and spiral computed tomography, which can detect a dilatation of the abdominal aorta. The AAA was defined as a focal dilation of the abdominal aorta at least 50% larger than the normal diameter. Patients with connective tissue disease, chronic renal failure, a known inflammatory or malignant disease, or familial AAAs were excluded from the study. A total of 436 age-matched control subjects (221 males and 215 females) from the same geographical area (Central Harbin) were recruited within the same period, and the aortic diameters of the control subjects were defined as a range from 19 to 22 mm.

Written informed consent was obtained from each participant prior to enrollment in the study. This study was approved by the Ethics Committee of Harbin Medical University and was conducted according to the principles of the Declaration of Helsinki.

### 2.2. DNA Isolation and Genotyping

Genomic DNA was isolated from whole blood samples from all of the patients and controls by TIANamp Blood DNA Kit (Tiangen Biotech, Beijing, China) according to the manufacturer's instructions and stored at −20°C.

The* RAGE* gene polymorphisms −374 T/A (rs1800624), −429 T/C (rs1800625), and G82S (rs2070600) were genotyped using the SNaPshot technique. The primers used were listed in Table 1. The genotyping was conducted using polymerase chain reaction (PCR) according to the manufacturer's protocol as it was described previously [21]. The experimental results were analyzed using GeneMapper 4.1 (Applied Biosystems, Foster City, CA, USA).

### 2.3. Enzyme-Linked Immunosorbent Assay (ELISA)

Blood samples were collected as soon as the diagnosis was established. The sRAGE levels in the sera were determined in duplicate using Quantikine sandwich ELISA kits (R&D Systems, Minneapolis, MN, USA) according to the manufacturer's instructions.

### 2.4. Assessment of the Aortic Diameter

The AAA size was determined as the greatest diameter of the infrarenal aorta using ultrasound. The maximum transverse and anteroposterior diameter of the infrarenal abdominal aorta was detected by an experienced vascular sonographer using a color Doppler ultrasound (GE Healthcare Technologies, Ultrasound, Milwaukee, WI, USA) with a 5 MHz transducer. The reproducibility of aortic measurements is regularly assessed in the vascular surgery department. An abdominal aortic aneurysm is defined as a dilation of the aorta of at least 1.5 times its normal diameter or greater than a 3 cm diameter.

### 2.5. Statistical Analyses

The statistical analyses were conducted using SPSS, version 19.0 (IBM, Armonk, NY, USA). The clinical data are expressed as the means ± standard deviation (SD) for the continuous variables and as the medians and percentage for the quantitative variables; a chi-squared test and Student's *t*-test were used to compare the variables between the two groups. The Hardy-Weinberg equilibrium (HWE) was tested using a chi-squared test to compare the observed genotype frequencies with the expected ones among the control subjects. Association between the polymorphism and the risk of AAA was evaluated using logistic regression analysis and was adjusted by age, gender, smoking, hypertension, diabetes mellitus, and dyslipidemia. The comparisons of the serum sRAGE levels among the different* RAGE* polymorphisms between the patients and controls were evaluated using Student's *t*-test for the normally distributed data or a Mann-Whitney *U* test for the nonparametric data. The associations of the gene polymorphisms with the aortic diameter were analyzed using a multiple linear regression model. The criterion for significance was set at *P* < 0.05 for all of the tests.

## 3. Results

### 3.1. Demographic Characteristics

The demographic characteristics of the participants are shown in Table 1. The mean age of the patients (292 males and 89 females) was 69.8 years (±8.1 years), compared with 70.2 years (±7.5 years) for the control subjects (221 males and 215 females). No significant differences were observed between the AAA patients and the controls with regard to age, diabetes, or hypertension. Significant gender and aortic diameter differences in the occurrence of AAA were observed. Compared with the controls, the AAA group included more smokers. Significant differences in the presence of dyslipidemia were also observed between the patients and controls.

### 3.2. Genotype and Allele Frequencies of the* RAGE* Polymorphisms in the AAA Patients and the Controls

The genotype and allele frequencies of the* RAGE* (−374 T/A, −429 T/C, and G82S) polymorphisms in the patients with AAA and the control individuals are shown in Table 2. A deviation from Hardy-Weinberg equilibrium for the* RAGE* polymorphisms was not found in the genotype distributions of the AAA patients and the control subjects (data not shown). The comparison of the genotype distributions between the AAA subjects and the control subjects using the *χ* ^2^ test revealed that there was a statistical association (*P* = 0.020) between the G82S polymorphism of* RAGE* and the risk for AAA (Table 2). In a dominant model (82GG + 82GS versus 82SS), no significant difference was detected between the AAA and control groups (*P* = 0.11). However, in a recessive model (82GG versus 82GS + 82SS), a significant difference was observed in the AAA group when compared with the controls (OR = 0.67, 95% CI: 0.50–0.88, *P* = 0.010). The prevalence of the* RAGE* 82S allele frequencies was significantly higher in the patients than in the controls (OR = 0.71, 95% CI: 0.57–0.88, *P* = 0.008). There were no statistically significant differences in the −374 T/A and −429 T/C genotypes and allele frequencies between the AAA cases and the control subjects (Table 2).

### 3.3. Associations between the* RAGE* G82S Polymorphism and the Demographic Characteristics

The associations between the* RAGE *G82S polymorphism and the demographic characteristics are shown in Table 3. In an analysis stratified by gender, an increased risk associated with the variant genotypes (82GS and 82SS) and alleles (82S) was found in the male patients (*P* = 0.025 for the genotype and *P* = 0.012 for the allele). When the sample was stratified according to smoking status, an increased risk associated with the variant genotypes and allele frequencies was more significant in the patients with AAA compared with the controls (*P* = 0.020 for the genotype and *P* = 0.008 for the allele). However, when the sample was stratified by either age, diabetes, or hypertension, no significant differences in the genotype or allele frequencies were detected between the AAA cases and the controls (*P* > 0.05) (Table 4).

### 3.4. The Serum Levels of sRAGE according to the* RAGE* Genotypes

**Figure 1:**
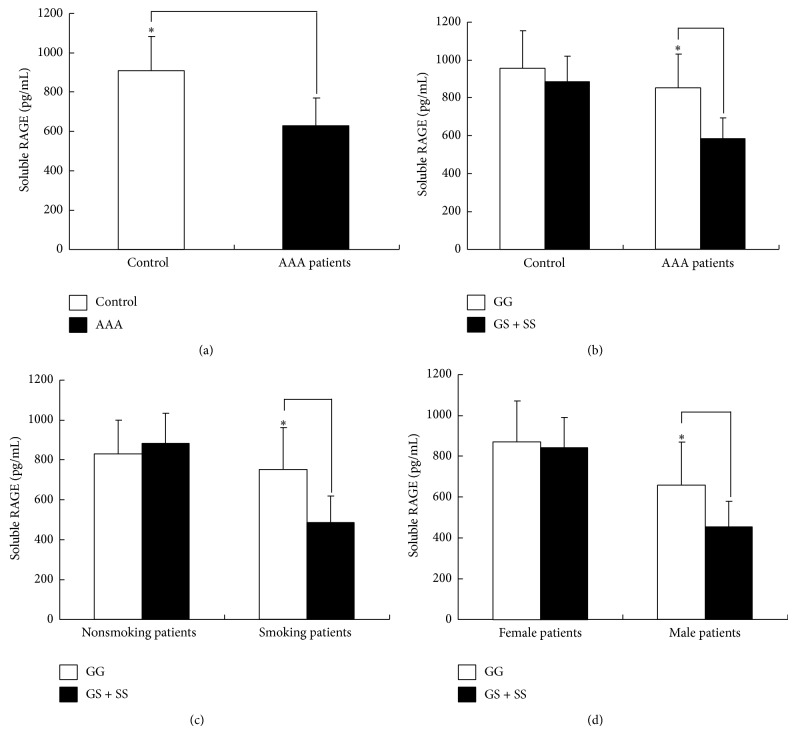
(a) The serum sRAGE levels in the AAA patients (*n* = 80) and the controls (*n* = 80). The blank box and the black box represent the serum sRAGE levels in the controls and the AAA patients, respectively. ^*^ *P* = 0.035 when comparing the serum sRAGE levels between the AAA patients and the controls. (b) The serum sRAGE levels in the AAA patients and controls stratified according to the* RAGE* genotype. The blank box represents the 82GG genotype, and the black box represents the 82GS + 82SS genotype. ^*^ *P* = 0.025. (c) The serum sRAGE levels in the AAA patients stratified according to smoking status and the* RAGE* genotype. ^*^ *P* = 0.012. (d) The serum sRAGE levels in the AAA patients stratified according to gender and the* RAGE* genotype. ^*^ *P* = 0.036. The serum sRAGE levels in the RRMS patients and the healthy individuals were measured using ELISA. The data are shown as the mean ± SD. An asterisk indicates *P* < 0.05.

The serum levels of sRAGE were measured in 80 AAA patients and 80 healthy controls. As shown in Figure 1, the serum sRAGE levels were significantly lower in the AAA patients than in the controls (918.58 ± 152.36 versus 625.39 ± 121.55 pg/mL, *P* = 0.035) (Figure 1(a)). Moreover, when the sample was stratified according to the* RAGE* genotypes, the serum sRAGE levels were significantly lower in the patients with the 82GS + 82SS genotype than in those with the 82GG genotype (852.25 ± 133.66 versus 586.32 ± 108.23 pg/mL, *P* = 0.025) (Figure 1(b)). However, in the healthy controls carrying the 82S allele, a slight but not significant decrease occurred. We also determined the serum RAGE levels in the AAA patients and controls who were stratified according to gender and smoking status. A significant decrease in the serum RAGE levels was found in the male patients (756.18 ± 117.15 versus 481.26 ± 128.88 pg/mL, *P* = 0.036) and the smoking patients (665.86 ± 109.33 versus 453.82 ± 115.72 pg/mL, *P* = 0.012) with the variant genotypes (82GG and 82GS) (Figures 1(c) and 1(d)).

### 3.5. Associations of the* RAGE G82S* Polymorphism with the AAA Aortic Diameter

**Figure 2:**
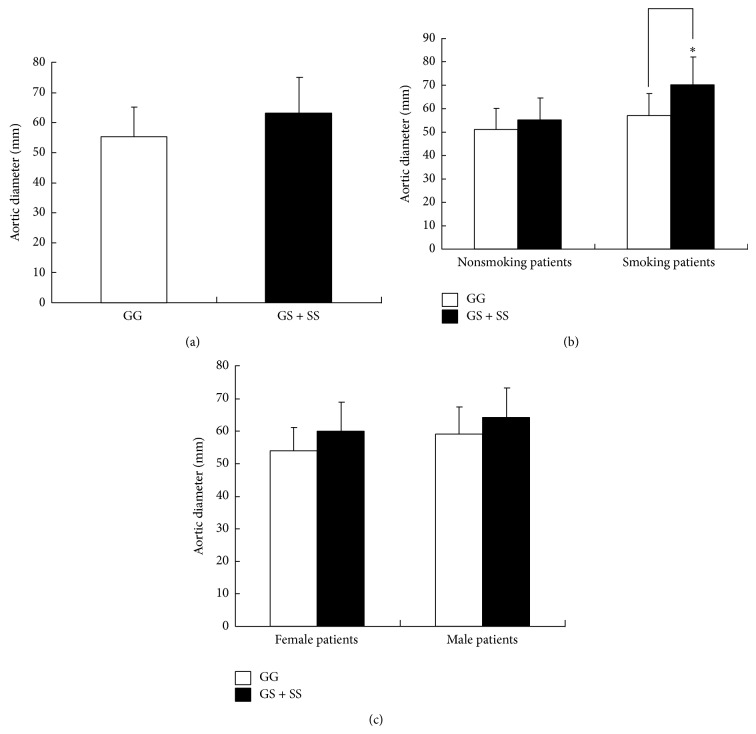
(a) The mean ± SD values of the AAA diameter stratified according to the presence of the mutated allele. (b) The mean ± SD values of the AAA diameter stratified according to smoking status and the presence of the mutated allele. ^*^ *P* = 0.044. (c) The mean ± SD values of the AAA diameter stratified according to gender and the presence of the mutated allele.

The associations of the* RAGE G82S* polymorphism with the AAA aortic diameter were explored, and the results are shown in Figure 2. The mean value of the AAA aortic diameter in the AAA patients with the variant genotypes (82GS and 82SS) was not significantly different from that of patients with the major 82GG genotype (55.8 ± 10.8 versus 63.2 ± 13.2 mm, *P* > 0.05) (Figure 2(a)). Interestingly, when the AAA patients were stratified by gender and smoking status, a significant increase in the aortic diameter was found in the smoking patients with the variant genotypes (82GS + 82SS) (57.8 ± 9.8 versus 70.2 ± 12.5 mm, *P* = 0.044) (Figure 2(b)). However, no difference in the aortic diameter was found in either the female or male patients with the variant genotypes (54.1 ± 8.2 versus 60.8 ± 9.3 in female and 59.6 ± 8.8 versus 64.5 ± 11.5 in male mm, *P* > 0.05) (Figure 2(c)).

## 4. Discussion

In this hospital-based, case-control study, we demonstrated for the first time that the G82S polymorphism of* RAGE* is associated with AAA. Further stratification revealed that men or smokers with the* RAGE* 82S allele may run a higher risk of acquiring AAA. Additionally, the serum sRAGE levels were significantly lower in AAA patients with the variant 82S allele than those carrying the major 82GG genotype.

As an inflammatory disease, the most significant pathologic feature of human AAA is the infiltration of inflammatory cells, including macrophages, monocytes, lymphocytes, and plasma cells, primarily to the outer part of the aorta [22]. Previous studies also demonstrated that oxidative stress is augmented during the process of AAA development [5]. Increased ROS is capable of enhancing inflammatory responses via the activation of NF-*κ*B, which is known to be a redox-sensitive transcription factor [23]. On the converse, activated inflammatory cells release ROS and further augment inflammatory responses. The engagement of RAGE with its ligands evokes the generation of intracellular ROS and results in the activation of MAPK and NF-*κ*B signaling, which then triggers the release of inflammatory factors, cytokines, and chemokines, thereby contributing to the development and progression of AAA [9]. Previous studies demonstrated that RAGE is highly expressed in the infiltrating macrophages of human aneurysm tissues [11].* In vivo *evidence also indicated that RAGE knockout mice are resistant to the formation of aneurysms [11], suggesting that RAGE signaling is involved in AAA development. The observations that RAGE is present in aortic aneurysm tissue and that its expression is significantly increased in aneurysms have confirmed its involvement in the pathogenesis of AAA. Despite these advances, the* RAGE* alleles that contribute to the pathology of AAA remain undiscovered.

Several large genome-wide association (GWAS) studies have been performed [24–26]; however, the results are rather inconsistent, and the reported odds ratios (ORs) for the identified risk alleles in these studies are very low. These discrepancies suggest that multiple risk loci are involved in the disease, and many are unidentified. In our case-control study, we show for the first time that the* RAGE* G82S polymorphism is associated with the risk of developing AAA. Our present findings imply that the G82S polymorphism of* RAGE* may serve as a genetic marker for predicting AAA in high-risk populations.

The pathogenesis of AAA is the result of interactions between genetic predispositions and environmental factors. Epidemiological surveys demonstrated that the male sex is a risk factor for AAA, and males run a four- to five-time greater risk than females [27]. Estrogen may play a protective role in AAA formation because estrogen was found to decrease inflammation and conversely correlate with MMP activity [28]. Being male is of course genetic, but it also affects environmental risk factors such as smoking, as well as hormonal levels, which are important mediators of aneurismal dilatation. In our present study, when the* RAGE* G82S genotype and allele frequency were further stratified by age, gender, smoking status, diabetes, and hypertension, an increased risk was found in the male and smoking subgroups of the AAA patients compared to the controls. Therefore, it is highly speculated that environmental risk factors, gender, and smoking may have interplay with the* RAGE* genetic predisposition in the present study.

The soluble form of RAGE (sRAGE) is a C-truncated secretory isoform of the receptor protein, which could work as a decoy receptor to abrogate RAGE signaling via inhibiting the binding of RAGE to its ligands. Therefore, the release of sRAGE may influence the regulation of RAGE-mediated functions in inflammatory events. The G82S polymorphism of* RAGE* results in glycine being changed to serine at position 82 of the third exon encoding the RAGE protein and has been shown to be associated with both reduced serum levels of sRAGE and increased sRAGE signaling compared with the more common G allele [19, 29, 30]. This change in sRAGE signaling affects downstream gene expression through MAPK and NF-*κ*B, both of which have been implicated in the inflammatory response in AAA [31]. In the present study, we showed that the serum levels of sRAGE were significantly decreased in AAA patients compared to the controls, supporting the potential role for the RAGE axis in the pathology of AAA. Moreover, we found that individuals carrying the mutated* RAGE* G82S S allele expressed lower sRAGE levels when compared with populations that carry the 82GG genotype. Given the key role of sRAGE in neutralizing circulating proinflammatory RAGE ligands, it is conceivable that the individuals with the 82S allele expressing reduced serum sRAGE levels may result in an increase in the amount of ligands, which may cause the subsequent cellular responses. Therefore, the individuals with the* RAGE* 82S allele will be more susceptible to RAGE ligand-induced inflammatory responses, which is a leading cause of AAA formation.

Accumulating epidemiological evidence confirmed that smoking is a significant contributing factor for AAA [32]. Current smokers have a significantly higher risk of developing AAA in both women and men than those who have never smoked [33]. Smoking also increases AAA growth, causing a greater risk of rupture and poor prognosis [34]. The formation and expansion of AAA involve widespread inflammation and extracellular matrix degradation. A recent study indicated that the AAA diameter was significantly associated with the hs-CRP plasma levels [35], which has evolved as an inflammatory risk marker of cardiovascular disease, suggesting that inflammation plays a potential pathogenic and causal role in the expansion of AAA. Other studies also suggested that an inhibitor against the platelet P2Y12 receptor reduces vascular inflammation and suppresses AAA expansion in* apoE−/−* mice [36]. These lines of evidence indicate that inhibiting inflammation may alleviate AAA expansion. Our study did not show any association of the polymorphisms with the aortic diameter; however, we confirmed a borderline association of the* RAGE* G82S polymorphism and the AAA diameter in smoking patients. Smoking as an environmental risk factor for AAA is very convincing and has been known for decades [32]. A study performed by UKSAT reported that active smoking is associated with an increased growth rate of 0.4 mm or 15%–20% greater than nonsmokers [34]. Additionally, cigarette smoke is an important exogenous source of reactive glycation products that can promote the formation of AGEs, thereby leading to RAGE-AGE axis-mediated signaling [37]. Based on the findings in our present study, it is conceivable that environmental exposure factors, in particular smoking, and genetic factors (82S allele of* RAGE*) may work synergistically in the pathophysiology of AAA expansion.

Despite the evidence, our study has several limitations that should be accounted for when interpreting the results. First, the study sample was not sufficiently large, which may lead to nonrepresentative results. Therefore, the preliminary findings of the present study are not definitive and must be replicated in a larger population. Second, selection bias in the patient or control populations cannot be entirely excluded. Additionally, the information regarding the important risk factors of AAA, such as smoking, was gathered from a self-reporting questionnaire, which may also introduce information bias. Third, other risk factors in the study group, such as* Chlamydia pneumoniae* infection and immune responses, which have been suggested to play a pivotal role in the pathogenesis of AAA, may have complicated the association between the* RAGE* polymorphisms and AAA. Finally, other functional polymorphisms located in the major histocompatibility complex locus may also affect the expression of sRAGE and contribute to the AAA risk, and their combined effects should not be neglected for predicting the occurrence, severity, and outcome of AAA. Larger patient and control cohorts from different ethnic backgrounds will be needed to confirm the association of the* RAGE* gene polymorphism with AAA in other populations.

In conclusion, our study is the first to show a significant association between the* RAGE* G82S polymorphism and the risk of developing AAA. Our findings support the notion that the G82S polymorphism of* RAGE* contributes to the development of AAA. In particular, males or smokers carrying the 82S allele of* RAGE*, which is associated with decreased sRAGE levels, may run a higher risk of developing AAA. Our study may provide clues for use in the evaluation of individual susceptibility to AAA and to explore effective measures for the control and prevention of AAA. However, this is a preliminary study, and the results need to be confirmed in a larger cohort.

## Figures and Tables

**Table 1:** Primers used in genotyping of RAGE −374 T/A, −429 T/C, and G82S polymorphisms.

*RAGE* polymorphisms	Direction	Primers
G82S (rs2070600)	Forward	5′-GCTGGGGTTGAAGGCTTTTTCT-3′
	Reverse	5′-CCGGACAGAAGCTTGGAAGGTC-3′

−374T/A (rs1800624) and −429T/C (rs1800625)	Forward	5′-CCCATCTTGATTGCGCAAAGTT-3′
	Reverse	5′-TCAAAAAACATGAGAAACCCCAGAA-3′

**Table 2:** Characteristics of AAA cases and controls.

Variables	AAA (*n* = 381)	Control (*n* = 436)	*P* value^a^
Mean age (years)	69.8 ± 8.1	70.2 ± 7.5	0.79
Male/female	292/89	221/215	<**0.001**
Aortic diameter (mm)	56.3 ± 7.5	21.2 ± 6.4	<**0.001**
Smokers, *n* (%)	206 (54.1)	145 (33.3)	<**0.001**
Diabetes, *n* (%)	89 (23.4)	86 (19.7)	0.23
Hypertension, *n* (%)	147 (38.6)	155 (35.6)	0.86
Dyslipidemia, *n* (%)	185 (48.6)	152 (34.9)	<**0.001**

Continuous data are presented as mean ± SD, median (range), or *n* (%)

^a^ *P* values under 0.05 were indicated in bold font.

**Table 3:** Genotype and allele frequencies of *RAGE *polymorphisms between AAA patients and controls and corresponding ORs for AAA.

Genotype and allele	AAA patients (*n* = 381)	Controls (*n* = 436)	OR (95% CI)	*P* value^a^
−374T/A (rs1800624)				
TT	282 (74.0)	303 (69.5)		0.31
TA	88 (23.1)	115 (26.4)		
AA	11 (2.9)	18 (4.1)		
TT + TA versus AA	370 (97.1)	418 (95.9)	1.45 (0.68–3.11)	0.34
TT versus TA + AA	99 (26.0)	133 (30.5)	1.25 (0.92–1.70)	0.15
T allele	652 (85.6)	721 (82.7)	1.00	
A allele	110 (14.4)	151 (17.3)	1.24 (0.95–1.62)	0.11

−429T/C (rs1800625)				
TT	297 (78.0)	344 (78.9)		0.85
TC	77 (20.2)	86 (19.7)		
CC	7 (1.8)	6 (1.4)		
TT + TC versus CC	374 (98.2)	430 (98.6)	0.75 (0.25–2.24)	0.60
TT versus TC + CC	84 (22.0)	92 (21.1)	0.95 (0.68–1.32)	0.74
T allele	671 (88.1)	774 (88.4)	1.00	
C allele	91 (11.9)	98 (11.6)	0.93 (0.69–1.27)	0.66

G82S (rs2070600)				
GG	187 (49.1)	258 (59.2)		0.020^b^
GS	155 (40.7)	149 (34.2)		
SS	39 (10.2)	29 (6.7)		
GG + GS versus SS	342 (89.8)	407 (93.3)	0.63 (0.38–1.03)	0.11
GG versus GS + SS	194 (50.9)	178 (40.8)	0.67 (0.50–0.88)	**0.010**
G allele	529 (69.4)	665 (76.3)	1.00	
S allele	233 (30.6)	207 (23.7)	0.71 (0.57–0.88)	**0.008**

Data are presented as number (%).

^a^Adjusted for age, gender, smoking, hypertension, diabetes mellitus, and dyslipidemia.

^b^ *P* values under 0.05 were indicated in bold font.

**Table 4:** A comparison between the baseline characteristics of the *RAGE* G82S genotypes and alleles in the AAA patient and control groups.

Characteristics	AAA patient group					Control group					*P*_G_^a^ value	*P*_A_^a^ value
	Genotype *n* (%)			Allele *n* (%)		Genotype *n* (%)			Allele *n* (%)			
	GG	GS	SS	G	S	GG	GS	SS	G	S		
Age												
≥70 years	103 (47.9)	95 (44.2)	17 (7.9)	301 (70.0)	129 (30.0)	136 (57.9)	84 (35.7)	15 (6.4)	356 (75.7)	114 (24.3)	0.11	0.052
<70 years	84 (50.6)	60 (36.1)	22 (13.3)	228 (68.7)	104 (31.3)	122 (60.7)	65 (32.3)	14 (7.0)	309 (76.9)	93 (23.1)	0.092	0.058
Gender												
Male	140 (47.9)	119 (40.8)	33 (11.3)	399 (68.3)	185 (31.7)	131 (59.3)	75 (33.9)	15 (6.8)	337 (76.2)	105 (23.8)	0.025^b^	0.012^b^
Female	47 (52.8)	36 (40.4)	6 (6.7)	130 (73.0)	48 (27.0)	127 (59.1)	74 (34.4)	14 (6.5)	328 (76.3)	102 (23.7)	0.59	0.40
Smoking												
Yes	91 (44.2)	95 (46.1)	20 (9.7)	277 (67.2)	135 (32.8)	87 (60.0)	50 (34.5)	8 (5.5)	224 (77.2)	66 (22.8)	**0.020**	**0.008**
No	96 (54.9)	60 (34.3)	19 (10.9)	252 (72.0)	98 (28.0)	171 (58.8)	99 (34.0)	21 (7.2)	441 (75.8)	141 (24.2)	0.37	0.20
Diabetes												
Yes	37 (41.6)	43 (48.3)	9 (10.1)	117 (65.7)	61 (34.3)	51 (59.3)	29 (33.7)	6 (7.0)	131 (76.2)	41 (23.8)	0.070	0.082
No	150 (51.4)	112 (38.4)	30 (10.3)	412 (70.5)	172 (29.5)	207 (59.1)	120 (34.3)	23 (6.6)	534 (76.3)	166 (23.7)	0.088	0.060
Hypertension												
Yes	69 (46.9)	62 (42.2)	16 (10.9)	200 (68.0)	94 (32.0)	91 (58.7)	54 (34.8)	10 (6.5)	236 (76.1)	74 (23.9)	0.093	0.056
No	118 (50.4)	93 (39.7)	23 (9.8)	329 (70.3)	139 (29.7)	167 (59.4)	95 (33.8)	19 (6.8)	429 (76.3)	133 (23.7)	0.10	0.069

*P*_G_: *P* value of the difference in alleles between the case and control groups; *P*_A_: *P* value of the difference in genotype between the case and control groups.

^a^Adjusted for age, gender, smoking, hypertension, diabetes mellitus, and dyslipidemia.

^b^ *P* values under 0.05 were indicated in bold font.
